# The Enemy within: Innate Surveillance-Mediated Cell Death, the Common Mechanism of Neurodegenerative Disease

**DOI:** 10.3389/fnins.2016.00193

**Published:** 2016-05-10

**Authors:** Robert I. Richards, Sarah A. Robertson, Louise V. O'Keefe, Dani Fornarino, Andrew Scott, Michael Lardelli, Bernhard T. Baune

**Affiliations:** ^1^Department of Genetics and Evolution, Centre for Molecular Pathology, School of Biological Sciences, The University of Adelaide Adelaide, SA, Australia; ^2^School of Paediatrics and Reproductive Health, Robinson Research Institute, The University of Adelaide Adelaide, SA, Australia; ^3^School of Medicine, Discipline of Psychiatry, The University of Adelaide Adelaide, SA, Australia

**Keywords:** dementia, innate autoimmunity, inflammation, neurodegeneration, Alzheimer's, Parkinson's, Huntington's

## Abstract

Neurodegenerative diseases comprise an array of progressive neurological disorders all characterized by the selective death of neurons in the central nervous system. Although, rare (familial) and common (sporadic) forms can occur for the same disease, it is unclear whether this reflects several distinct pathogenic pathways or the convergence of different causes into a common form of nerve cell death. Remarkably, neurodegenerative diseases are increasingly found to be accompanied by activation of the innate immune surveillance system normally associated with pathogen recognition and response. Innate surveillance is the cell's quality control system for the purpose of detecting such danger signals and responding in an appropriate manner. Innate surveillance is an “intelligent system,” in that the manner of response is relevant to the magnitude and duration of the threat. If possible, the threat is dealt with within the cell in which it is detected, by degrading the danger signal(s) and restoring homeostasis. If this is not successful then an inflammatory response is instigated that is aimed at restricting the spread of the threat by elevating degradative pathways, sensitizing neighboring cells, and recruiting specialized cell types to the site. If the danger signal persists, then the ultimate response can include not only the programmed cell death of the original cell, but the contents of this dead cell can also bring about the death of adjacent sensitized cells. These responses are clearly aimed at destroying the ability of the detected pathogen to propagate and spread. Innate surveillance comprises intracellular, extracellular, non-cell autonomous and systemic processes. Recent studies have revealed how multiple steps in these processes involve proteins that, through their mutation, have been linked to many familial forms of neurodegenerative disease. This suggests that individuals harboring these mutations may have an amplified response to innate-mediated damage in neural tissues, and renders innate surveillance mediated cell death a plausible common pathogenic pathway responsible for neurodegenerative diseases, in both familial and sporadic forms. Here we have assembled evidence in favor of the hypothesis that neurodegenerative disease is the cumulative result of chronic activation of the innate surveillance pathway, triggered by endogenous or environmental *danger* or *damage associated molecular patterns* in a progressively expanding cascade of inflammation, tissue damage and cell death.

## Introduction

Different chronic neurodegenerative diseases are distinguished by the type of neurons and the regions of the brain that are first affected. In each case the different initial symptoms and functions lost are due to the progressive death of specific neurons. Neurodegenerative diseases also tend to be diverse in their timing, with some being congenital whereas others are late age-at-onset. Those that progress, gradually exhibit commonalities. A large number and wide variety of causes of neurodegeneration have been identified. These range from inherited single gene mutation effects, to environmental factors or complex mixtures of both. It has been difficult to establish cause and effect pathways and to reconcile how so many apparently disparate conditions all result in similar outcomes. Either the diverse causes trigger multiple pathogenic pathways, or alternatively they must converge into a common mechanism of cell death and tissue damage. Familial cases of neurodegeneration, while typically less frequent than sporadic cases, have provided opportunities to gain insight into the genetic factors and thereby the underlying biological processes.

In this review we present evidence to support the hypothesis that a common pathogenic mechanism for neurodegenerative disease exists, and is mediated by innate surveillance-cell death.

## Overview

Cell death is vital in the biology of multicellular organisms. It is integral to tissue remodeling during development, where programmed cell death is required to deconstruct, remove and replace tissues. Cell death is also a vital *defense strategy* in combating infection. Where cells harbor and are unable to destroy pathogens then cell death is a means to limit propagation and spread of the pathogen. Similarly, if a cell has acquired somatic mutations that predispose to tumorigenesis, then elimination by cell death is a means to neutralize the threat. In each of these situations the innate immune surveillance system programs the appropriate cell death pathways.

In the last few years, a growing body of literature has appeared, pointing to innate surveillance system activity being an early, elemental and consistent hallmark of neurodegenerative disease. In particular recent genome wide association studies in Alzheimer's disease (see Table 1A) and genetic modification studies in animal models of various neurodegenerative diseases (see Table 1B) have identified multiple components of the innate surveillance system as genetic determinants. These findings are inconsistent and difficult to reconcile with the prevailing theories of neurodegenerative disease. There is now decisive evidence supporting the hypothesis that innate surveillance mediated cell death is a common cause, not simply a consequence, of nerve cell death and therefore the principle causal mechanism of neurodegenerative disease.

**Table 1:** **Innate surveillance hallmarks in human neurodegenerative disease and their animal models**.

**Disease**	**IS component(s)**	**References/review**
**(A) INNATE SURVEILLANCE COMPONENTS IN HUMAN NEURODEGENERATIVE DISEASE**		
AD	“Innate immune”	see Bettens et al., 2013; Zhang et al., 2013
	Glial cell activation	see Mosher and Wyss-Coray, 2014
	Astrocytosis	see Rodriguez-Vieitez et al., 2016
	Various (by GWAS)	Escott-Price et al., 2014
ALS	Cytokines & immune	Batra et al., 2016
AGS	Interferon	Crow, 2015; Rice et al., 2012, 2014
BSN	Interferon	Livingston et al., 2014
DM1, DM2	Interferon	Rhodes et al., 2012
HD	IL-4, 6, 8, 10, TNF	Bjorkqvist et al., 2008
Multiple	XBP-1	Dunys et al., 2014
Multiple	Microglial priming	see Perry and Holmes, 2014
PD	Microglia	Russo et al., 2014; Schapansky et al., 2015
SP	Interferon	Crow et al., 2014
**(B) INNATE SURVEILLANCE MODIFIERS/MARKERS IN ANIMAL MODELS OF NEURODEGENERATIVE DISEASE**		
**Disease**	**Modifier/marker**	**References**
AD	NLRP1	Tan et al., 2014
AD	CD33/TREM2	Chan et al., 2015
AD	CSF1R	Olmos-Alonso et al., 2016
AGS (ADAR1)	MDA-5 (IFIH1)	Liddicoat et al., 2015; Pestal et al., 2015
ALS (SOD1)	XBP-1	Hetz et al., 2009
	TLR4	Lee et al., 2015
FTD (toll, CHMP2B)	Serpin5	Ahmad et al., 2009
PD (chemical)	HMG-B1	Sasaki et al., 2016
PD (LRRK2)	TLR4	Moehle et al., 2012
HD, SCAs	TNF, drosomycin	Samaraweera et al., 2013
SCA6	MyD88	Aikawa et al., 2015

The innate surveillance and immune inflammatory response system is a complex, highly integrated means of surveillance, detection and response to pathogens and precancerous cells. The current understanding of this system is almost certainly incomplete and is the composite from studies in multiple species and cell types that may well differ from one another in detail (Beutler et al., 2007; Buchan et al., 2014; Liu et al., 2014; Pellegrino et al., 2014). In the following sections we review the components and attributes of this system from a perspective that sets a context for its potential role in neurodegenerative disease pathogenesis.

## The innate immune inflammatory response to pathogen recognition

### Innate immune surveillance system

Biological competition comes in various forms. In the host-pathogen “arms race” a key requirement for host survival is the ability to sense danger and respond appropriately. Integral to this is the ability to distinguish “self” from “non-self.” Such recognition can be direct, by virtue of molecular patterns that are either intrinsic or foreign to the host, or indirect, reflecting the consequences of foreign intrusion, such as changes in homeostasis or stress responses.

Activation occurs via one or more *Pattern Recognition Receptors* (*PRR*s) that detect trigger molecules—foreign *Pathogen Associated Molecular Patterns* (*PAMP*s) or endogenous *Danger Associated Molecular Patterns* (*DAMP*s), the latter typically being elevated in response to perturbation of homeostasis. The immediate response is an escalation of appropriate degradation pathways (e.g., autophagy) in order to eliminate the intruder and/or restore homeostasis. Persistence of trigger signal molecules provokes the production and release of pro-inflammatory and anti-inflammatory cytokines, which sensitize adjacent cells. This is followed by programmed death (by necroptosis) of cells in which trigger molecules are detected (Wu et al., 2012; Kaczmarek et al., 2013; Pasparakis and Vandenabeele, 2015). Cell death via necroptosis causes release of cell contents that are recognized as *Damage Associated Molecular Patterns* (*dAMPs*) by adjacent sensitized cells (Lu et al., 2014). In-turn, this expands the foci of cells undergoing PRR-triggered innate surveillance-mediated cell death. Cytokines also recruit specialized cells to the focal response site, contributing to its amplification, then ultimately containment and resolution (Vanha-aho et al., 2015).

The innate surveillance system is ancient, having an integral role in the biology of animals and plants. Its origins probably trace back to bacteria where the defense system of restriction-modification enabled bacteria to identify their own DNA and selectively degrade that of an intruder. In plants and animals the post-transcriptional modification of nucleic acids is common (over 100 different types of modification to tRNAs alone). At least some of this modification appears devoid of function, other than to identify nucleic acids as belonging to the host. Indeed, such modifications are required for exogenous mRNA to avoid stimulating the innate surveillance response, even when its sequence is identical to that of the endogenous mRNA (Warren et al., 2007; Andries et al., 2013).

The selective pressure of the host-pathogen “arms race” has seen this system both evolve rapidly and reach into most, if not all, cellular compartments such that it monitors the surveillance of a vast array of cellular processes. At least in some organisms, certain elements participate in developmental processes. It is this system that has also evolved to detect and respond to detrimental consequences of somatic DNA mutations, such as those that can increase the risk of neoplasia (Feng and Martin, 2015). Since such mutations accumulate and are more likely to cause dysfunction over time, there is good reason to think that longer-lived animals are dependent upon this system to minimize the occurrence of cancer, particularly later in life (Caulin and Maley, 2011). In this regard it is noteworthy that this system appears to increase in sensitivity with age (von Bernhardi et al., 2010).

The innate surveillance system therefore confers major biological benefits in the front line of defense in detecting and responding to pathogens and also in the monitoring and removal of rogue endogenous cells that can threaten the host through malignancy. These benefits come at a cost—in particular the risk of bringing about the inappropriate death of host cells, such as that seen in neurodegeneration and other examples of inflammatory damage.

This raises the question of why some individuals and not others are more susceptible to neurodegeneration at different phases of the life course. It seems likely that disposition is conferred by intrinsic errors in the system itself, i.e., mutations in relevant genes, and/or threshold responses to environmental factors that are detected as “danger” signals—each contributing to cumulative risk of neurodegeneration. Importantly, the genetic causes of neurodegenerative disease have led to the identification of innate surveillance mediated cell death as the proximal cause of, at least certain forms of, neurodegeneration. Many of the genetic changes linked with neurodegeneration can be demonstrated to specifically escalate the innate surveillance pathway, as detailed in later sections of this review. First, it is relevant to navigate the various cellular compartments and systems that trigger the innate surveillance response.

### Innate surveillance—mediated cell death

#### Sensing—pathogen and quality control sentinels

The first characterized components of the innate surveillance system, *Toll* and *spätzle*, were initially identified for their role in *Drosophila* development (Stein and Nüsslein-Volhard, 1992). Subsequent demonstration (Lemaitre et al., 1996) that this receptor-ligand combination also mediates antifungal responses revealed that the innate surveillance system is not simply a host-pathogen detection-response system, but is also integral to normal biological processes. This functional diversity reflects the capacity for innate surveillance to recognize specific endogenous trigger molecules as well as foreign molecules that signal a danger to the cell. Characterization of *spätzle* also revealed that activation steps are sometimes invoked, to switch the pattern of a signal molecule to one that is recognized by the relevant receptors (Mizuguchi et al., 1998). More recently it has become clear that the role of this surveillance system in development includes cellular elimination based on cell competition and fitness (Meyer et al., 2014).

The sensing of pathogens occurs both directly and indirectly in both the extracellular and intracellular compartments, the latter as an extension of mechanisms that monitor and maintain homeostasis. While quality control mechanisms likely exist for all cellular compartments—that of the endoplasmic reticulum unfolded protein response is best understood (Gardner et al., 2013).

##### Endoplasmic reticulum stress unfolded protein response

The endoplasmic reticulum (ER) interacts dynamically with membranous structures in the cell including the Golgi apparatus, mitochondria, peroxisomes, endosomes, lysosomes, and autophagosomes. The ER plays a key role in protein biosynthesis of cellular components and therefore it is a target for pathogens seeking to co-opt this capacity for replication, as well as being a risk site for the synthesis of aberrant proteins that can contribute to tumourigenesis. Newly synthesized proteins entering the ER are either folded into their required conformation for targeting to organelles or identified as unfolded and are then destined for degradation.

The unfolded protein response (UPR) monitors and maintains protein-folding homeostasis within the ER (Gardner et al., 2013; Senft and Ronai, 2015). Yeast experiments demonstrate that this ancient system comprises sentinel sensors (Gardner et al., 2013). In the ER, proteins that exceed the protein-folding capacity activate three stress sensor proteins—IRE1α, PERK, and ATF6. At least one of these sentinels has evolved to trigger the innate surveillance system. IRE1 is present in all eukaryotes and conveys the UPR response to the innate surveillance system. IRE1α cleaves endogenous XBP1 mRNA to remove an intron, enabling the resultant mRNA to encode the active form of the XBP1 transcription factor, that in turn stimulates expression of proteins involved in restoration of proteostasis, as well as cytokine synthesis (Zeng et al., 2010). Cleavage of the XBP1 intron causes the RNA to acquire an unusual 3′ cyclic phosphate that enables its recognition as a DAMP by RIG-I, a cytosolic PRR of the innate surveillance system (Cho et al., 2013; Eckard et al., 2014). Recent studies reveal that this intron RNA is an endogenous DAMP in Aicardi-Goutieres Syndrome, a congenital neurodegenerative disease caused by mutation in one of a number of nucleic acid metabolizing enzymes (Eckard et al., 2014). Communication between ER components and the innate surveillance system is further exemplified in Unc93-B, an ER resident membrane protein that is indispensable to TLR3, TLR7, and TLR9 signaling (Brinkmann et al., 2007; Nakano et al., 2010).

##### Mitochondrial stress unfolded protein response

A distinct system of unfolded protein response in the mitochondria is best characterized in *C. elegans* (Haynes and Ron, 2010; Pellegrino et al., 2014). Mitochondrial function is essential for homeostasis and regulation of apoptosis. At least one mechanism exists for sensing mitochondrial dysfunction. The transcription factor ATFS-1 is normally imported into the mitochondria, through the TOM/TIM import complex, where it is degraded by the resident Lon protease. Disruption to this mitochondrial import causes cytoplasmic accumulation, then nuclear localization of ATFS-1, resulting in the expression of mitochondria-specific protein folding machinery. An intriguing gate-keeper of this mitochondrial import process is *TOMM40*, the gene for which is located within the *APOE* block of linkage disequilibrium that confers susceptibility to Alzheimer's Disease (Roses et al., 2010), as discussed later. Some, but not all of the factors identified in *C. elegans* have orthologs in humans indicating that additional components are likely to participate in this process. Indeed PINK1 has been identified in humans as a protein that in normal cells is partially imported into the mitochondria, via the TOM/TIM complex. In dysfunctional mitochondria this import is inhibited, resulting in an excess of PINK1 accumulation in the outer membrane where it recruits the ubiquitinating enzyme Parkin that then initiates mitophagy (Harbauer et al., 2014). The details of this pathway for the removal of dysfunctional mitochondria have recently been elucidated (Celardo et al., 2014). Loss-of-function mutations in either PINK1 or Parkin are the cause of familial cases of Parkinson's Disease (OMIM 608309 and 602544) implicating defects in this pathway, in the pathogenesis of this form of neurodegeneration.

#### Trigger molecules

**Figure 1:**
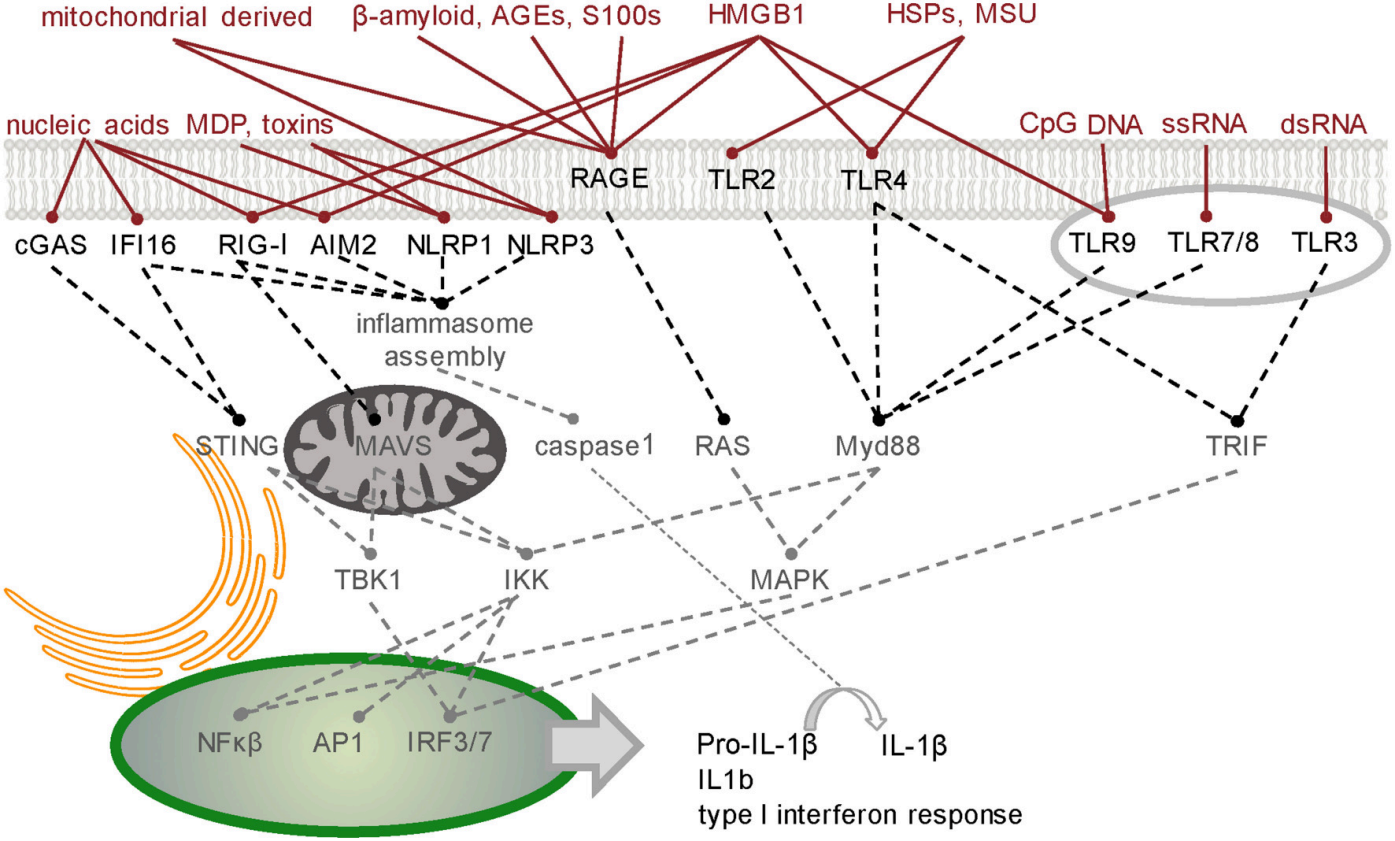
**Simplified schematic of signaling junctions that integrate the recognition of pathogen, host or environmentally derived DAMPs and dAMPs by their cognate receptors**. Viral or host derived nucleic acids are recognized by several PRR families according to distinct structural characteristics and their location. cGAS generates second messenger cGAMP to activate STING-dependent signaling (Hornung et al., 2014). Other cytoplasmic DNA sensors, such as IFI16, converge on this pathway (Goubau et al., 2010). RIG-I has dual functions in inducing nuclear translocation of transcription factors such as NF-κB and IRF-3/7 through MAVS (Yoneyama et al., 2016) and MAVS-independent inflammasome activation (Poeck et al., 2010). Like RIG-I, inflammasome forming PRRs AIM2 and NLRP1/3 direct procytokine conversion by activating caspase-1 (Kim et al., 2016; Thaiss et al., 2016; in response to stimuli such as bacteria-derived MDP or toxins (Wen et al., 2013). Transcription factors NF-κB, AP-1, and IRF-3/7 can also translocate to the nucleus following activation of the endosomal TLRs which signal through either Myd88 or TRIF. Membrane bound TLR2/4 innervate these pathways on detecting host derived dAMPs such as HSPs and MSU (Gelderblom et al., 2015). Binding of pro-inflammatory ligands including AGEs, S100/calgranulin, amphoterin (HMGB1), and amyloid beta-peptide to RAGE, the receptor of advanced glycation end-products, triggers an increase in proinflammatory molecules, oxidative stressors and cytokines (Ray et al., 2015). HMGB1 is a diverse PRR activator (Harris et al., 2012). TLR3-mediated necrosis is via TRIF (Kaiser et al., 2013). A table of DAMPs/dAMPs and their respective PRRs is included in Supplementary Section. PRR, pattern recognition receptor; cGAS, cyclic guanosine monophosphate-adenosine monophosphate synthase; STING, stimulator of IFN genes; IFI16, IFN-Υ-inducible protein 16; RIG-I, retinioc acid inducible gene-I; NF-κB, nuclear factor kappa B; IRF, interferon regulatory factor; MAVS, mitochondrial antiviral signaling protein; AIM2, absent in melanoma 2; NLRP, NOD like receptor, pyrin domain containing 1 and 3; MDP, muramyl-dipeptide; AP1, activator protein 1; TLRs, Toll-like receptors; Myd88, myeloid differentiation primary response 88; TRIF, Toll/interleukin-1 receptor domain containing adapter-inducing interferon-β; DAMPs, danger-associated molecular patterns; dAMPs, damage-associated molecular patterns; AGEs, advanced glycation end products; HSPs, heat-shock proteins; MSU, monosodium urate.

The host defense system operates in an environment comprising a complex mixture of molecules - some pose a threat while others are either harmless or beneficial. Recognition of threatening molecules occurs on the basis of their molecular pattern (Figure 1).

##### Pathogen associated molecular patterns

Molecular patterns exhibited by a diverse array of exogenous, foreign pathogen molecules of viral, bacterial and fungal origin are recognized by the innate immune surveillance system and trigger an inflammatory response. Some “non-pathogenic” molecules also act as trigger molecules, invoking a sterile inflammatory response. Such molecules may be threatening, such as asbestos (Hamilton, 1980; Rock et al., 2010) and therefore inflammation may aid in their removal. In general the distinction between exogenous and endogenous molecules is clear, however there are very important exceptions.

##### Danger associated molecular patterns (DAMPs – see box 1 regarding nomenclature)

In addition to pathogen-derived molecules, there are a variety of endogenous agents that can act as triggers of the innate surveillance response system. For endogenous agents the distinction between “self” and “non-self” typically involves modification of the molecular structure. RNA molecules are a good example, with a multitude of modified forms, many of which don't affect function, but some of which distinguish “self” from “non-self.” A good example of this distinction is seen in experiments of “induced pluripotent stem cells”. Fibroblasts can be transformed into induced pluripotent stem cells by transfection with four mRNAs encoding the required factors. However these mRNAs need to be subjected to RNA modification beforehand otherwise they will elicit an inflammatory response in the fibroblasts (Warren et al., 2007). Such modifications can occur via enzymes that recognize specific sequence motifs or appear at the RNA 5′ or 3′ends as a result of the type of enzymatic cleavage of the RNA. For example, when activated as part of the Unfolded Protein Response, IRE-1 cleaves not only XBP-1 mRNA (as described above) but other RNAs as well, producing a pool of RNA products with unusual 3′ cyclic phosphates, that trigger a “danger” response (Eckard et al., 2014). In this manner endogenous RNA molecules are recruited to act as signaling molecules in the innate surveillance system (Eckard et al., 2014).

Box 1 **Danger (*DAMP*) and Damage (*dAMP*) Associated Molecular Patterns.**

While labeled in the literature with common and therefore confusing acronyms, two types of molecular patterns factor in the innate surveillance system—those that signal ***danger***, and those that arise from cell ***damage*** (the latter via specific forms of cell death). In an effort to distinguish these, we have used “D” for danger and “d” for damage.

Pattern recognition is a major element of innate surveillance. While the host needs to be able to distinguish “self” from “non-self,” and do so in a manner uncorrupted by pathogen mimicry, it also needs to monitor endogenous trigger molecules—those that act as sentinels of a developmental switch or a perturbation in homeostasis. Post-transcriptional modification of RNA and post-translational modification of proteins are processes that cells utilize to either mask or unmask molecular patterns that trigger recognition. **Pathogen Associated Molecular Patterns (PAMPs)** can be molecules derived from pathogens that either contain recognizable patterns or are devoid of modifications that would otherwise mask their recognition. Pathogen RNAs will not have been exposed to the same post-transcriptional modifications as host RNAs and can be distinguished on this basis (Nallagatla et al., 2008). Furthermore, some nuclease cleavage of RNA exposes structures that are recognized by Pattern Recognition Receptors (e.g. RIG-I-mediated recognition of viral RNA bearing 5′-diphosphates (Goubau et al., 2014). In this manner, endogenous RNAs can also be recognized as **Danger Associated Molecular Patterns (DAMPs)** and utilized as intracellular danger signaling molecules. Ribonuclease IRE-1 cleavage produces a pool of RNA products with unusual 3′ cyclic phosphates, that are recognized by Pattern Recognition Receptors.

Cell death comes in various forms, certain types of which lead to the extracellular appearance of normally intracellular molecules. Some of these cell damage molecules—**damage Associated Molecular Patterns (*dAMPs*)—**are also recognized by PRRs. Viruses that lyse cells or environmental damage (such as ischemic stroke or head injury) can thereby activate the innate surveillance response. Forms of programmed cell death (such as necroptosis) facilitate the release of dAMPs that are recognized by PRRs on adjacent cells. Sensitized adjacent cells can have elevated levels of PRRs thereby mediating their non-cell autonomous death. In this manner dAMPs act as extracellular signaling molecules.

##### Damage associated molecular patterns (dAMPs—see box 1 regarding nomenclature)

Non-autonomous cell death is part of the strategy to restrict the spread of a persistent pathogen. Indeed the finding that *spätzle* is an extracellular ligand for the cell surface receptor *Toll*, is indicative of cell-cell signaling in the system (Mizuguchi et al., 1998). Intracellular molecules released from one cell can act as extracellular trigger molecules for the innate surveillance response in another cell. Cell damage that results in release of damage associated molecular patterns (*dAMPs*) can include environmental causes (Bernard et al., 2012). Endogenous damage molecules include RNAs (Bernard et al., 2012) and proteins such as HMG-B1 (Kim et al., 2006; Harris et al., 2012; Gelderblom et al., 2015) and F-Actin (Ahrens et al., 2012). These *dAMPs* are recognized by members of the *Toll*-like Receptor family (TLRs). TLRs have, at least in some cases, been induced in the cells adjacent to the primary cell undergoing an inflammatory response, by virtue of the cytokines that the primary response cell produces (Khoo et al., 2011). This localized sensitization creates a zone or field of cells that are able to detect products shed from the dead primary cell.

#### Sentinels (pattern recognition receptors, PRRs)

PRRs are the detectors or sentinels of the innate surveillance system. They are sensors of an alarm system that not only detects intruders but also danger signals that cells make to amplify a local response to disturbed homeostasis. PRRs typically act synergistically rather than in isolation (Nasirudeen et al., 2011). A diverse multitude of trigger molecules are detected by PRRs, via recognition of distinct patterns in the trigger molecules (e.g., the presence or absence of a chemical modification). This recognition occurs in intra- and extra-cellular compartments and is mediated by families of related receptor proteins (Figure 1).

##### Toll-like receptors (TLRs)

*Toll* was discovered in *Drosophila* as a key regulator of both development and host defense against pathogens. *Toll* is a transmembrane receptor and binds the extracellular ligand *spätzle* in its developmental role. *Toll* is related in structure to the cytokine receptors and is one of a family of such proteins, comprising 9 members in *Drosophila* (Valanne et al., 2011)and 13 in humans (Roach et al., 2005). Sequence comparison does not reveal clear orthologous relationships—they are therefore termed *Toll*-like receptors (TLRs) in vertebrates and *Toll*-related receptors (TRRs) in *Drosophila*. Functional relationships are likely based on their cellular localization, some common pattern recognition and inter-connected signal transduction pathways (Figure 1). This inter-connected signal transduction of TLRs suggests that they function collectively as a network, to mount an integrated response. TLRs sample the extracellular environment and the interstices of endosomes, where they recognize PAMPs and dAMPs. Endosomes can fuse with autophagosomes (Klionsky et al., 2016), therefore endosomal TLRs are also exposed to the internal constituents of autophagosomes, such as RNA stress granules and defective mitochondria, that have been targeted for degradation.

##### Other pattern recognition receptors (RLRs, NLRs, and CLRs)

At least three other families of Pattern Recognition Receptors have been identified and characterized to varying extents (Figure 1). The most thoroughly studied of these are the RIG-I-like Receptors (RLRs) (Loo and Gale, 2011). They are located in the cytoplasm and recognize both exogenous PAMPs and endogenous DAMPs. The functional extent of this family is uncertain, as there are no clear orthologs in some species. For example in *Drosophila* the closest ortholog for the characterized human RLRs is *Dicer 2* and there is good evidence that *Drosophila Dicer 2* does indeed act as an intracellular PRR, in addition to its role in miRNA biosynthesis (MacKay et al., 2014). Two additional families of proteins, Nod-like Receptors (NLRs) and C-type lectin receptors (CLRs), also act as PRRs (see references to Figure 1), but the mechanisms by which they activate the innate surveillance response are not well defined. Some PRRs may be specific to certain specialized cells that are recruited to the site of pattern recognition detection by the early response extracellular signaling molecules (Applequist et al., 2002).

#### Early response mechanisms

**Figure 2:**
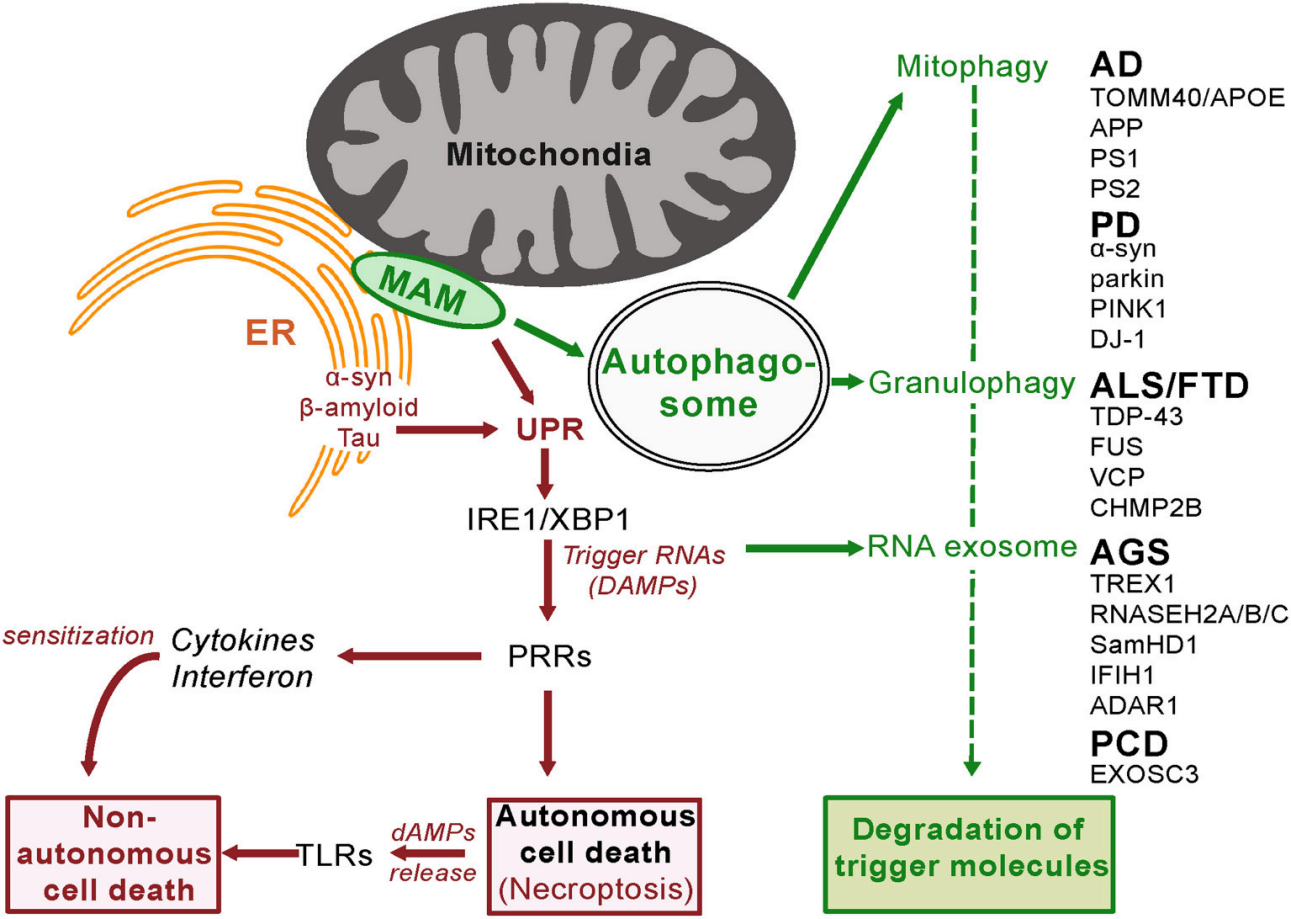
**Innate Surveillance Pathways and Genetic Contributions to Neurodegenerative Disease**. In response to activation by trigger molecules, innate surveillance recruits a diverse array of degradative processes to reduce the danger signal(s) and restore homeostasis. Genetic lesions in one or other of these degradative pathways will lead to a build-up of danger signal (DAMP) levels beyond a threshold that elicits an escalated response. Pattern Recognition Receptors (PRRs) orchestrate the release of signaling molecules directly (cytokines and interferon) and indirectly (damage-associated molecular patterns, dAMPs, from programmed necrotizing cells) leading to sensitization and eventual non-autonomous death of adjacent cells. It is noteworthy that the ER stress response is activated by amyloid beta in brain cells (Fonseca et al., 2013).

The immediate response to activation of innate surveillance appears aimed at degrading the danger signal(s) and restoring homeostasis by reprogramming gene expression. In some cases this includes the cessation of processes that contribute to the trigger signal and increasing the activity of degradative pathways. A diverse array of such degradative processes are activated (Box 2, Figure 2).

Box 2 **Cellular Clearance Mechanisms Implicated in Innate Surveillance Response.**

**Pathways responsible for proteostasis and RNAstasis in the event of stress response**

**Unfolded Protein Response (UPR)**—integrated pathway for sensing and overcoming the accumulation of unfolded or mis-folded proteins within the ER, or in the event of persistent accumulation, to induce innate surveillance-mediated cell death (Senft and Ronai, 2015). Involves 3 distinct activation pathways—ATF6/ATF6f; PERK/ATF4; IRE1/XBP1s. IRE1 cleavage product of XBP-1 mRNA is recognized by the cytosolic pattern recognition receptor RIG-I of the innate surveillance system.

**Endoplasmic Reticulum Associated Degradation (ERAD)**—pathway is modulated by the UPR and targets misfolded proteins to the cytosol for their ubiquitination and subsequent proteosomal degradation.

**Autophagy**—degradation of macromolecular complexes including protein aggregates and RNA granules, as well as damaged organelles through fusion with lysosomes.

**Granulophagy**—in response to stress polysomes disassociate and their mRNAs form granules (stress granules or P-bodies) with specific RNA binding proteins (including TDP43 and FUS). These stress granules hold the RNAs until the stress is relieved or failing this are transported (by VCP) to autophagosomes that then fuse with lysosomes (regulated by the inhibitor CHMP2B) for degradation and subsequent recycling.

**Mitochondrial Unfolded Protein Response (UPR**^*mt*^**)**—the location of activating transcription factor associated with stress-1 (ATFS-1) is sensitive to unfolded protein stress inside the mitochondria. Stress restricts mitochondrial importation of ATFS-1 resulting in higher cytosolic level, that in turn leads to its nuclear localization where it activates target genes, the products of which act to protect the mitochondria. Failure to resolve the mitochondrial stress leads to autophagy (also known as **mitophagy**) of the damaged mitochondria.

**Heat Shock Response (HSR)**—includes dissociation of heat shock factor-1 (HSF1) from inhibitory binding with heat shock factor 90 (HSP90). HSF-1 induces heat shock target genes that in turn mediate protein quality control pathways.

**Nonsense Mediated Decay (NMD)**—a highly conserved RNA integrity surveillance pathway that not only leads to the elimination of faulty endogenous transcripts and viral RNAs, but also regulates levels of ~10% of normal cellular transcripts (Rigby and Rehwinkel, 2015).

**Ubiquitin-Proteosome System (UPS)**—the major pathway for non-lysosomal degradation of proteins.

**RNA exosome** (*SKIV2L*)—an exoribonuclease complex for RNA processing, quality control and turnover regulation. In low stress environments it efficiently digests mRNAs including those encoding cytokines. It is negatively regulated by stress, resulting in greater stability of cytokine mRNAs (and therefore elevated cytokine levels) as well as incomplete removal of IRE1 RNA digestion products that are recognized by RIG-Like Receptors (RLRs) of the innate surveillance system (e.g., XBP-1 RNA fragments that are recognized by RIG-I) resulting in its activation (Blin and Fitzgerald, 2015). The *SKIV2L* RNA exosome limits the activation of RIG-I-like receptors (Eckard et al., 2014). Loss-of-function mutations in the *EXOSC3* gene, encoding the RRP40 RNA exosome protein, are a cause of the inherited progressive neurodegenerative disorder pontocerebellar hypoplasia (Wan et al., 2012).

##### Autophagy—in its multiple forms

The destruction of molecules deemed dangerous makes good use of the cell's normal recycling mechanism, autophagy. The activity of autophagy varies above a basal state in response to various stimuli including innate surveillance activation. Cross-talk between the ER unfolded protein response, autophagy and mitochondria underlies the cellular response to stress (Senft and Ronai, 2015), with autophagosomes formed at ER-mitochondria contact sites (Hamasaki et al., 2013). There are multiple sub-types of autophagy dependent upon the materials being recycled (Klionsky et al., 2016).

##### Granulophagy

In response to stress, cells reprogram translation—they disassociate polysomes from mRNA and specific proteins are aggregated into granules (Stress Granules and P-bodies; Buchan et al., 2013). If the stress is transient and relieved then these granules disaggregate, the mRNA is subject to polysome reassembly and translation resumes. If the stress stimulus continues then the granules are trafficked to an autophagosome that then fuses with a lysosome—a vesicle containing degradative enzymes. The contents of these autolysosomes are then degraded to the building blocks for *de novo* transcription of genes, including those that facilitate the cell's response to the stress stimulus. The genes for four proteins involved in this process (*TDP43, FUS, VCP*, and *CHMP2B*) have mutations in various familial cases of the neurodegenerative diseases fronto-temporal dementia (FTD) or amyotrophic lateral sclerosis (ALS). While TDP43 and FUS have multiple functions, both have been found in RNA stress granules (Bentmann et al., 2013; Li et al., 2013). Genetic screens in yeast have identified *CDC48* (the ortholog of human protein VCP) as a regulator of the trafficking of these aggregates for degradation (Buchan et al., 2013). Mitochondrial function in neuronal cells depends on VCP/Cdc48-mediated quality control (Fang et al., 2015). Loss-of-function mutations in VCP have been detected in some familial cases of ALS and FTD (Talbot and Ansorge, 2006). CHMP2B also has multiple functions but one of these is an inhibitor of autophagosome-lysosome fusion. Gain-of-function mutations in *CHMP2B* are also found in familial cases of ALS and FTD (Talbot and Ansorge, 2006). Loss of function mutations in *SamHD1*, a regulator of stress granule assembly, are another cause of the neurodegenerative disease known as Aicardi-Gouitieres Disease (Hu et al., 2015 *and see below*). This clustering of mutations around the autophagy function implies that defective stress granule metabolism has a causal role to play in neurodegenerative disease.

##### Mitophagy

Cellular metabolism of glucose occurs as a balance between glycolysis and oxidative phosphorylation. Glycolysis in the cytoplasm produces biosynthetic building blocks (e.g., pyruvate), whereas oxidative phosphorylation in the mitochondria produces energy (ATP). Once cells cease dividing and differentiate they have reduced biosynthetic requirements and shift to mainly utilizing glucose as an energy source for cell function. Since pathogens strive to replicate themselves, their biosynthesis requires structural components and therefore a reduction in mitochondrial metabolism, in favor of glycolysis, facilitates their replication. Similarly cancer cells favor glycolysis as it facilitates cell division. The state of the mitochondria and its perturbation is therefore closely monitored, including the mechanism of mitochondrial unfolded protein response (described above). The functional integrity of the mitochondria is maintained by mitophagy, a specific form of autophagy. Damaged or dysfunctional mitochondria are recognized as such and shunted into the autophagy process, for recycling and regeneration. Defects in this process are detected by PRRs to trigger alarm in the cell, resulting in an elevated response to the danger, including increased mitophagy (Pellegrino and Haynes, 2015). If increased mitophagy is unsuccessful in resolving the dysfunction then ultimately the complete destruction of the cell is programmed (Yu et al., 2015).

The benefit of mitochondrial-regulated innate immunity is that it provides resistance to pathogen infection (Pellegrino and Haynes, 2015). However there is also a cost in that loss of function mutations in proteins that are required for mitophagy will lead to inappropriate cell death. As discussed above, it is noteworthy that mutations in two such proteins *parkin* and *PINK1*are known causes of autosomal recessive early onset Parkinson Disease (Kitada et al., 1998; Piccoli et al., 2008; Figure 2). Impaired autophagy is also a hallmark of Alzheimer's disease (Salminen et al., 2013).

Other forms of autophagy almost certainly exist, indeed such a mechanism involving the nucleus has just been described (Dou et al., 2015), widening the scope of autophagy as part of the innate surveillance monitoring and response system.

##### Cytokines—sensitization permissive of focal cell death

Induction of intracellular and extracellular signals is another element of the innate surveillance response. The innate surveillance response includes the production of extracellular signaling molecules (collectively termed cytokines, including chemokines, interferons, interleukins, and tumor necrosis factor). These molecules have been identified in different cell types and model organisms and likely vary in composition and function in different circumstances (e.g., Beutler et al., 2007). They act as both feed-forward and feed-back controls, enabling a mixture of responses and at varying distances, either in the trigger cell, its neighbors or systemically.

Interferons comprise several families of structurally related proteins that exhibit a wide range of biological activities (Pestka et al., 2004). Type I interferons are an essential component of the brain's innate immune defense, conferring protection against viral infection (McGlasson et al., 2015). Interferons are both inducers of TLRs and are induced by activation of TLRs (Khoo et al., 2011) and clearly act to amplify a danger response. Similarly, tumor necrosis factor is required for pathogen resistance in the CNS (Francisco et al., 2015) and acts in sensitizing cells to programmed death pathways.

Mechanisms are also in place to limit the duration, spread and extent of response. Negative feed-back controls include miRNAs specific to the induced mRNAs (Zhou et al., 2011) and ribonucleases (Regnase-1 and Roquin) specific to structures found in cytokine mRNAs (Mino et al., 2015). Regnase-1 and Roquin act in different compartments of the cell and also serve to increase degradation of RNA P-bodies and stress granules.

#### Persistent response mechanisms—programmed cell death

In the early activation phase of innate surveillance, cytokine release will create a zone of sensitized or primed cells that then enables a focal response (Khoo et al., 2011). If the trigger persists, a major integrated defense strategy is activated to ensure not only the original cellular site of danger molecule detection is eliminated, but also adjacent cells in the vicinity will undergo non-autonomous cell death. This focal response restricts the pathogen's ability to survive, propagate and spread. Similarly, rogue cells and perhaps their adjacent siblings or daughter cells, that have accumulated somatic mutations and therefore begun the process of tumorigenesis, can be eliminated by a zone of cell death around the primary trigger of innate surveillance activation. As part of this process the manner of cell death has an important role to play.

Given the importance of programmed cell death as a defense mechanism and the intense selective pressure this system faces from rapidly evolving pathogens, it is hardly surprising that multiple forms of programmed cell death have evolved. There appear to be two functionally distinct forms with respect to the innate surveillance system—(1) those that release *dAMPs* and/or pro-inflammatory cytokines to perpetuate and spread the response, and (2) those that do not release *dAMPs*, and thereby limit and contain the response.

***Apoptosis*** is a well-characterized form of programmed cell death (Elmore, 2007). It is utilized in normal developmental processes as well as host-pathogen defense and typically involves the degradation of cellular components commensurate with cell death, either internally or by surrounding cells. Apoptosis is therefore not normally associated with the release of *dAMPs*. The molecular mechanism involves a cascade of activated caspases. Caspases are broadly classified as initiator (caspase-2, 8, 9, 10), effector (caspase-3, 6, 7) or inflammatory (caspase-1, 4, 5). The intrinsic pathway of caspase activation involves mitochondria. A variety of stimuli cause mitochondria to release cytochrome *c*, that in turn binds to and sequesters the adaptor protein Apaf-1. Apaf-1 binds, aggregates and cleaves procaspase-9 molecules, to trigger a caspase cascade (Elmore, 2007). While apoptosis is the canonical form of programmed cell death and has certain conserved features, there are clearly variant forms that may differ between cell types, species or have specific roles.

***Pyroptosis*** is a distinct form of cell death that may be restricted to only certain immune cell types and also may vary between such cell types (Bortoluci and Medzhitov, 2010; Nyström et al., 2013). These cells may either be resident or have been attracted to the site of infection by chemokines released by the initial responding cell. Such cells are activated through a specific set of cytoplasmic PRRs, the Nod-like receptors (NLRs)—although the mechanism of this activation is as yet unclear (Harijith et al., 2014). These dying cells can in turn release pro-inflammatory cytokines to amplify the response. For example NLRP3-inflammasome activating *dAMPs* stimulate an inflammatory response in glia that contributes to brain inflammation after injury (Savage et al., 2012).

***Necroptosis*** is another distinct form of programmed cell death (Christofferson and Yuan, 2010; Wu et al., 2012). In order to bring about the non-autonomous cell death of adjacent sensitized cells, a distinct form of extracellular communication occurs. *dAMPs* that are released upon the death of the trigger cell act as activators of PRRs on adjacent sensitized cells. *dAMPs* may include those originating from pathogens, but it is clear that certain endogenous molecules are also able to act in this capacity. Endogenous *dAMPs* include heat shock proteins (HSPs), the nuclear protein HMGB1, F-Actin, RNA, and DNA (Kim et al., 2006*;* Ahrens et al., 2012; Shichita et al., 2014; Gelderblom et al., 2015). Various TLRs are specific receptors for *dAMPs*. Intriguingly, HSPs and HMGB1 are also thought to mediate inflammatory activation in ischemic stroke (Kim et al., 2006; Gelderblom et al., 2015). It is noteworthy that anti-HMGB1 antibody has recently been found to exert neuroprotection in a rat model of Parkinson's disease (Sasaki et al., 2016). Furthermore, Murakami et al. (2014) found that necroptosis, not apoptosis, is a key mediator of cell loss and dAMP-mediated inflammation in a mouse model of dsRNA-induced retinal degeneration.

**“*Other”-optosis*** encompasses the fact that some organisms, such as *Drosophila*, do not have orthologs for essential components of pyroptosis or necroptosis (i.e., inflammasome constituents or RIP1 and RIP3) yet they do exhibit *Toll*-like receptor mediated cell death. This is due to the expression of intracellular *dAMPs* (Samaraweera et al., 2013) indicating that there are additional cell death pathways that lead to the release of *dAMPs*, given the requirement of *TLR*s for activation by extracellular signals. One such candidate pathway is that mediated by Poly(ADP-Ribose) Polymerase (PARP-1) (Wu et al., 2012). PARP-1 links nuclear and mitochondrial function and is conserved in *Drosophila*. Dysfunction of mitochondria is a key step in PARP-1-induced cell death, although the mechanism of PARP-1 induced mitochondria dysfunction remains unknown. Reactive oxygen species have frequently been linked to both inflammation (Harijith et al., 2014) and neurodegenerative disease and it is noteworthy that over-expression of superoxide dismutase (SOD) protects against mitochondrial-initiated PARP-mediated cell death (Kiningham et al., 1999). Kauppinen et al. (2011) demonstrated a role for PARP in microglial responses to amyloid beta, suggesting a role in the pathogenesis of Alzheimer's disease (Martire et al., 2015).

### Neurodegenerative diseases—causes and correlations

The ever-growing emergence of one or more components of the innate surveillance system in neurodegenerative pathogenesis implicates this cell system as a common mechanism for neurodegenerative disease (Tables 1, 2). Remarkably, plausible mechanisms can be postulated by which each of the known genetic causes of neurodegeneration feed into one or other form of activation of the innate surveillance system. Moreover, the genetic lesion as proximal trigger shows how activation of the innate surveillance system is not simply a bystander effect, but a central upstream cause of pathogenesis. These rare genetic causes of disease can help delineate the responsible pathogenic pathway for the more common, sporadic forms of the disease. Furthermore, identification of responsible genes enables the establishment of genetic animal models with which to define and dissect the pathogenic pathway from mutation to symptoms.

**Table 2:** **Summary of evidence for role of innate immunity in the etiology of distinct forms of neurodegenerative disease**.

**Disease**	**Pathways or proteins involved in innate immunity**						
	**UPR/MAM**	**ER Stress**	**XPB-1**	**CHMP2B**	**VCP**	**IRG**	**IL**
AD	+^a^^,^ ^b^	+^c^	+^d^				
PD			+^d^				
FTD/ALS			+^d^^,^ ^e^	+^f^^,^ ^g^	+^h^		
HD^*^			+^d^		+^i^	+^j^	+^k^
AGS			+^l^			+^m^	

*IRG, interferon regulated genes; IL, interleukins;*

* *, and other expanded repeat diseases*.

*References:*

a *Cornejo and Hetz, 2013,*

b *Lee et al., 2010,*

c *Fonseca et al., 2013,*

d *Dunys et al., 2014,*

e *Hetz et al., 2009,*

f *Cox et al., 2010,*

g *Ahmad et al., 2009,*

h *Azuma et al., 2014,*

i *Higashiyama et al., 2002*

j *Rhodes et al., 2012,*

k *Bjorkqvist et al., 2008,*

l *Eckard et al., 2014,*

m *Rice et al., 2014*.

#### Genetic

Mendelian causes of neurodegeneration roughly fall into two distinct groups exhibiting either recessive inheritance due to loss-of-function mutations, or dominant transmission typically due to some form of gain-of-function.

***Loss-of-function*** mutations inform on mechanisms of pathophysiology, and also are instructive to indicate those pathways that are candidates for sporadic forms of the disease. Amyotrophic lateral sclerosis (ALS) and frontotemporal dementia (FTD) exhibit overlap in both clinical and genetic features. They share multiple common genetic causes and can be found separately or co-incidentally in different members of the same family (Lattante et al., 2015). The majority of cases of ALS and FTD appear not to have a genetic cause and therefore must have environmental origins. Genetic causes for ALS and FTD can be loss of function mutations in a variety of proteins including TDP43, FUS, and VCP. Until recently, a link between these proteins has been difficult to pinpoint but remarkably, it is now apparent that all of these proteins share the ability to interact with RNA. TDP43, and FUS are both components of RNA stress granules, while VCP is required for the trafficking of these granules to autosomes. Reasonably, it can be seen that loss of this degradative pathway can cause inflammation and ultimately neurodegeneration by elevating endogenous RNA “danger” signal(s)—*DAMPs*.

Evidence for RNA degradation as a key step in control of the innate surveillance pathway also comes from the loss-of-function mutations that cause Aicardi–Goutières Syndrome (AGS). AGS is a genetically heterogeneous autosomal recessive encephalopathy characterized in its most severe form by cerebral atrophy, leukodystrophy, intracranial calcifications, chronic cerebrospinal fluid (CSF) lymphocytosis, increased CSF alpha-interferon (IFNA1), and negative serologic investigations for common prenatal infections. AGS is phenotypically similar to *in utero* viral infection. Severe neurologic dysfunction becomes clinically apparent in infancy, and manifests as progressive microcephaly, spasticity, dystonic posturing, profound psychomotor retardation, and often death in early childhood. Loss-of-function mutations in genes in at least six distinct loci are able give rise to the constellation of symptoms that defines Aicardi–Goutières Syndrome. Four of these (*RNAseH2A, RNAseH2B, RNAseH2C*, and *ADAR1*) are in genes that encode RNA-metabolizing proteins. The remaining two genes that have been identified (*TREX1* and *SAMHD1*) also encode enzymes that have roles in nucleic acid metabolism, and have recently both been shown to also have ribonuclease activity (Ryoo et al., 2014; Yuan et al., 2015). Deficiency in any one of these six enzymes is thought to result in the accumulation of endogenous nucleic acids that are sensed as “non-self” by RIG-I-like receptors, that in turn activate innate inflammatory pathways (Crow and Rehwinkel, 2009; Hofer and Campbell, 2013; Rigby and Rehwinkel, 2015), indeed AGS has been termed an “interferonopathy” (Crow, 2011). The XBP-1 intronic RNA generated by IRE-1 cleavage has been identified as an endogenous danger RNA in the pathogenesis of AGS (Eckard et al., 2014). A more general role for RNA degradation in preventing neurodegeneration is indicated by loss-of-function mutations in the RNA exosome component EXOSC3, that are a cause of Pontocerebellar Hypoplasia (PCH1) (Wan et al., 2012; Rigby and Rehwinkel, 2015). The RNA exosome has a key role in innate sensing of RNA regulating cytokine production (Blin and Fitzgerald, 2015).

Therefore, loss-of-function mutations in a variety of proteins that are required for the degradation of RNA identify the accumulation of specific RNA danger molecules that trigger innate surveillance activation as a common causal mechanism through which neurodegeneration is initiated.

***Gain-of-function*** mutations in neurodegenerative diseases have generally proven more difficult to attribute. In a few cases the gain-of-function can be an enhancement of the normal function of the relevant protein and a plausible pathway to neurodegeneration is evident. For example, CHMP2B is an inhibitor of autophagosome-lysosome fusion (West et al., 2015). Therefore, dominant ALS/FTD causing mutations that enhance inhibitory action of CHMP2B reduce the degradative capacity of this pathway, decreasing the turn-over and causing a build-up of danger associated molecular patterns (including trigger RNAs). Similarly, dominant AGS-causing mutations in IFIH1 (MDA5) enhance its affinity for RNA *DAMPs*, with consequent increased activation of downstream interferon signaling and innate surveillance-mediated cell death.

In other cases, the pathophysiological pathway from the gain-of-function mutation to clinical symptoms remains evasive. There are approximately 20 dominantly inherited neurodegenerative diseases caused by expanded repeat mutations. The genes in which these repeats are located are neither structurally nor functionally related, so either there are many different pathogenic pathways or the gain-of-function responsible is not an enhancement of the normal functions of these proteins. Some proteins harbor the expanded repeat within regions encoding polyglutamine, and the resulting accumulation of polyglutamine has long been investigated as a possible common toxic agent. However, this is difficult to reconcile with observations that cells containing polyglutamine aggregates have a survival advantage (Sisodia, 1998) and, at least in the case of huntingtin protein, polyglutamine is substantially less toxic within the context of the remaining protein (Barbaro et al., 2015). A distinct form of repeat associated non-AUG translation has been described in which polypeptides are translated from all reading frames of repeat RNA in the absence of an initiator AUG. These polypeptides have also been found in patients with expanded repeat diseases but again do not correlate well with the sites of pathology (Mann, 2015; Mackenzie et al., 2015). In addition, some diseases (ALS and FTD) have multiple genetic causes in addition to an expanded repeat disease locus. Cases of ALS/FTD that aren't due to repeat expansion cannot feasibly share an expanded polypeptide repeat. The only way to explain these conflicting observations is to propose distinct pathogenic pathways for these diseases or otherwise a single pathogenic pathway, independent of expanded polypeptide toxicity.

***RNA pathogenesis*** is one pathogenic pathway that accommodates all of the existing evidence. Recognition of expanded repeat RNA as an innate surveillance trigger molecule is an alternative potential common mechanism for neurodegeneration, since in all cases of dominantly inherited expanded repeat disease, the expanded repeat DNA sequences are transcribed into RNA.

A precedent for RNA as the causal pathogenic agent in expanded repeat diseases has been proposed in myotonic dystrophy (DM). This disease is due to expansion of either of two repeat sequences. These repeats are transcribed as either 3′ untranslated RNA (CUG in DM1) or intronic RNA (CCUG in DM2). Both of these expanded repeat RNAs bind and sequester the alternative splicing factor *muscleblind* and as a consequence, alter the balance of alternative splicing of multiple genes in the muscle. The muscle-related component of the disease phenotype can be modeled in mice by either muscle-specific expression of the repeat RNA (Mankodi et al., 2000) or by reduction in endogenous muscleblind protein (Kanadia et al., 2003), providing clear evidence in support of expanded repeat RNA being the causal pathogenic agent.

An additional mechanism for RNA pathology in expanded repeat diseases has come from *Drosophila* models where RNA in various forms has been tested for its ability to initiate cell death (McLeod et al., 2005; Lawlor et al., 2011; van Eyk et al., 2011; Yu et al., 2011; Samaraweera et al., 2013). Of these, double strand CAG.CUG expanded repeat RNA was clearly found to cause cell death (Lawlor et al., 2011; Yu et al., 2011). All dominantly inherited expanded repeat disease loci exhibit bi-directional transcription (Batra et al., 2010), so double-strand expanded repeat RNA is plausible as a common cause in these diseases. In the *Drosophila* model, expanded repeats of CAG.CUG_100_ were cleaved to CAG_7_mers. These CAG_7_mers have also been detected in Huntington's Disease brain RNA (Banez-Coronel et al., 2012). Components of the innate surveillance pathway were subsequently shown to be required for this form of cell death in *Drosophila* (Samaraweera et al., 2013). Innate surveillance activation is evident, through the elevation in expression levels of its key targets *drosomycin* and cytokine *eiger* (ortholog of tumor necrosis factor). These hallmark pathogen response changes in *Drosophila* are consistent with observations of immune pathway activation (increased circulating levels of IL-4, IL-6, IL-8, IL-10, and TNF) before clinical onset in Huntington's disease Bjorkqvist et al. (2008). Mutant huntingtin protein does not affect the intrinsic phenotype of human Huntington's Disease T lymphocytes (Miller et al., 2015) supporting the notion that innate surveillance activation is responsible for the observed increase in cytokine activity, rather than the adaptive immune system. In the cataracts of either DM1 or DM2 individuals, up-regulated genes are highly enriched in both interferon-regulated genes (IRGs) and genes associated with response to dsRNA (Rhodes et al., 2012), demonstrating the RNA-triggered activation of innate surveillance in at least this symptom of myotonic dystrophy pathology.

Further evidence of a causal role for innate surveillance in expanded repeat disease comes from the finding that a mouse model of SCA6 can be modified by MyD88, a major adaptor protein in the conveyance of TLR signaling (Aikawa et al., 2015). SCA6 is another expanded CAG repeat disease thought to be caused by expanded polyglutamine. Histological analysis of the cerebellum of this knock-in mouse model showed the predominance of M1-like pro-inflammatory microglia and this was concomitant with elevated expression levels of tumor necrosis factor, interleukin-6, Toll-like receptors 2 and 7.

Until recently an expanded repeat at a single locus was typically the sole identified mutation in these dominantly inherited expanded repeat diseases. However, an exception to this general rule is the genetically heterogeneous disease ALS/FTD. Some inherited cases of ALS/FTD are due to expansion of a 6 base repeat in the intron of the C9orf72 gene, others are due to non-repeat mutations in a variety of other genes. The functions of these genes therefore give insight into how a single pathogenic mechanism might be responsible. Animal models for mutations in ALS/FTD genes have been instructive of the molecular pathway of pathogenesis. A variety of mutations in SOD1 cause ALS and a mouse model for one of these (SOD^G93A^) exhibits a phenotype consistent with the symptoms of the human disease. The survival of SOD^G93A^ mice is extended by genetic removal of the TLR4 pattern recognition receptor, indicating a role for innate surveillance in the pathogenesis (Lee et al., 2015).

The normal function of ALS/FTD causing genes is instructive. As discussed previously, loss-of-function mutations occur in *TDP-43* and *FUS* components of RNA stress granules (Ito and Suzuki, 2011) and in *VCP*, required for trafficking of the granules to the autophagosome (Azuma et al., 2014). Gain-of-function mutations in *CHMP2B* also cause ALS and this protein acts as an inhibitor of autophagosome-lysosome fusion (Cox et al., 2010). Together these observations suggest that defects in granulophagy are a likely cause of ALS/FTD. As detailed above, various forms of autophagy are critical for the degradation of *DAMPs*, including those of endogenous origin. Therefore, a clear path for the activation of innate surveillance is again evident, as is the potential for pathogens or environmental stress to contribute to the etiology of these diseases by independently activating the immune surveillance pathway.

Expansion of a GGGGCC repeat in the intron of the C9orf72 gene is a major cause of inherited ALS/FTD, adding further weight to the argument for RNA being the best candidate for a common trigger molecule in expanded repeat neurodegenerative diseases. Indeed expanded GGGGCC repeat RNA does cause neurodegeneration in a *Drosophila* model (Xu et al., 2013). The pathogenic mechanism responsible for expanded GGGGCC repeat RNA to cause ALS/FTD is as yet unclear however recent reports indicate that key regulators of nucleocytoplasmic transport are compromised in *Drosophila* models (Freibaum et al., 2015; Zhang et al., 2015). The GGGGCC repeat RNA is retained in the nucleus not only in these *Drosophila* models but also in cells derived from ALS/FTD affected individuals. In ALS/FTD affected individuals the nucleolus exhibits stress (Haeusler et al., 2014) and it will be of interest to see whether this stress mediates the recently described nuclear autophagy (Dou et al., 2015).

***Parkinson's Disease*** (PD) is a progressive neurodegenerative disease that is thought to have both genetic and environmental causes. Certain chemicals in the environment are strongly associated with PD. These include the herbicides rotenone and paraquat (Tanner et al., 2011). Rotenone is an inhibitor of mitochondrial complex I, while paraquat is a cause of oxidative stress. Mitochondrial dysfunction and oxidative stress are both thought to be elements of PD pathogenesis and are consistent with the rare genetic causes of the disease. The chemical 6-hydroxydopamine has been used to establish rat models of PD taking advantage of the specific accumulation of this toxin into catecholaminergic neurons (Simola et al., 2007). A role for innate surveillance in PD has recently been revealed in this rat model by the finding that an antibody directed against the dAMP HMG-B1 is neuroprotective (Sasaki et al., 2016).

Multiple genetic loci have been identified for familial cases of Parkinson's Disease that can segregate in either a dominant or recessive manner, most likely indicating gain- or loss-of-function respectively. The responsible genes and their mutations have been identified at many but not all loci and include *SNCA, PARK2, PINK1, LRRK2, DJ-1*. Loss-of-function mutations in either *PARK2* (encoding parkin) or *PINK1* cause early onset recessive PD, with both proteins having important roles to play in mitophagy (as described earlier). *Drosophila* models reveal that mitochondrial dysfunction in *PINK1* mutants is complemented by *parkin* (Park et al., 2006) and that *parkin* mutations lead to dopaminergic neuron loss, that can be rescued by increased glutathione S-transferase activity (Whitworth et al., 2005). Furthermore, *Drosophila parkin* mutants exhibit elevated biomarkers of innate immune responses in pathogenesis (e.g., *Diptericin* protein), indicating the activation of innate surveillance (Greene et al., 2005). Finally, *LRRK2* functionally interacts with *Parkin, DJ-1*, and *PINK-1* (Vendorova et al., 2009).

The relationship between these proteins and others, wherein mutant forms cause PD, has been unclear (Schapansky et al., 2015). However, Norris et al. (2015) recently report convergence of Parkin, PINK1, and α-synuclein in stress-induced mitochondrial remodeling. *SNCA* encodes α-synuclein and mutations in this gene were the first described in familial cases of PD. α-synuclein is also found in the protein aggregates known as Lewy Bodies that are found in the brains of some, but not all PD patients. The PD-causing *SNCA* mutations occur on a single allele and can be either missense mutations or increases in gene copy number, implicating a gain-of-function mechanism in the contribution of *SNCA* to disease pathogenesis. Expression of human α-synuclein in *Drosophila* neurons recapitulates symptoms of PD including loss of motor control, development of neuronal inclusions and degeneration of dopaminergic neurons. This phenotype is again rescued by expression of *parkin* indicating commonality in pathogenesis (Haywood and Staveley, 2004).

α-synuclein is not located in the mitochondria itself but in the mitochondria-associated membrane (MAM), a distinct compartment of the endoplasmic reticulum (Guardia-Laguarta et al., 2014; Figure 2). Disease-causing point mutations in α-synuclein reduce its association with the MAM implicating this localization in disease pathology. The MAM is the site of autophagosome formation (Hamasaki et al., 2013) and therefore a key component of a major degradative control pathway for the innate surveillance response. In a further indicator of commonality of pathogenesis, Ye et al. (2015) recently reported Parkin-mediated mitophagy in mutant hAPP neurons and Alzheimer's disease patient brains.

Gain-of-function mutations in the LRRK2 gene are responsible for the most frequent inherited cause of PD, although with low disease penetrance of 32% at 80 years of age (Goldwurm et al., 2007) suggesting interaction with other genetic and/or environmental factors. LRRK2 has multiple functions including roles in autophagy and mediating microglial proinflammatory responses (Moehle et al., 2012; Schapansky et al., 2014). These relationships have led to the conclusion that inflammation has a causal role to play in Parkinson's Disease (Russo et al., 2014).

***Alzheimer's Disease (AD)*** occurs in both sporadic and familial forms, with genetic causes or contributions to each form. Familial forms are earlier onset, with mutations found in genes that encode the amyloid precursor protein (APP) and two components of its cleavage (presenilin 1 and 2) to amyloid beta protein, a major component of amyloid plaques found in AD brain. These findings led to the amyloid cascade hypothesis—in essence that fragments of APP cause AD (Schellenberg and Montine, 2012; Tanzi, 2012; Hardy et al., 2014; Masters et al., 2015). This hypothesis has prevailed over the field of AD research for more than 25 years (Hardy and Higgins, 1992). Failure to progress to effective prevention or treatment of AD has provoked a recent call to focus on testing alternative hypotheses (Herrup, 2015).

One competing hypothesis involves the pathogenic interference with axonal transport of neurofilament and neurotubule proteins (Iqbal et al., 1977), subsequently identified as the microtubule associated protein tau (Grundke-Iqbal et al., 1986). The brains of individuals with AD exhibit abnormal phosphorylation and aggregation of tau into paired helical filaments in the form of neurofibrillary tangles.

Amyloid beta plaques and neurofibrillary tangles are the two main histopathological hallmarks of AD. Tau is encoded by a single gene *MAPT*, that is mutated in some cases of fronto-temporal dementia and certain other neurodegenerative diseases (Iqbal et al., 2005) but somewhat surprisingly, not in AD (Iqbal et al., 2015). Attempts to fuse these two hypotheses include proposals that the hallmark features of pathology directly influence one another (Mudher and Lovestone, 2002), for example the concept of “tauopathies” where aberrantly folded proteins (including tau, amyloid beta and α–synuclein) lead to the formation of toxic structures that spread through the brain, in a similar manner to prions (Goedert, 2015). The contribution of such structures to pathology has been challenged given the lack of correlation between plaque load and cognitive impairment in AD, with alternative forms of amyloid beta proposed as the neurotoxic agent (Muller-Schiffmann et al., 2016). So while unifying the field to some extent, this fused hypothesis has not provided an agreed mechanism of toxicity, nor does it explain additional genetic causes of, and risk factors for, Alzheimer's disease.

Like numerous other neurodegenerative diseases, increased inflammation correlates with Alzheimer's disease progression and has therefore been proposed as a disease mechanism (Heneka and O'Banion, 2007; Heneka et al., 2014, 2015). However since inflammation has been observed in a diverse array of neurodegenerative diseases, with seemingly distinct causes, this has led to the view that inflammation is a protective response to neurodegeneration, rather than a cause of it. Recently, genome-wide association studies of late-onset Alzheimer's disease have however identified susceptibility variants in loci harboring innate immune-related genes, including *CLU, CR1, CD33, EPHA1, MS4A4E/MS4A6A, PTK2B, TREM1, TREM2*, and *TREML2* (see Perry and Holmes, 2014). Several of these proteins have now been found to functionally interact (Chan et al., 2015). Therefore, elements of the innate surveillance and response system are indeed rate-limiting for Alzheimer's disease pathogenesis. Intriguingly, these are proteins more typically associated with microglia than neurons (Perry and Holmes, 2014), consistent with a non-cell autonomous mechanism for neurodegeneration. Indeed microglial dysfunction is a hallmark of the aging brain and Alzheimer's disease (Mosher and Wyss-Coray, 2014).

##### Conclusions regarding genetic causes of neurodegeneration

Together these observations form a compelling case that the innate surveillance system is a central upstream cause of neurodegenerative disease. The complexity of causal agents—whether genetic or environmental—can be explained by convergence around elevated susceptibility or active triggering of inflammation through the innate surveillance pathway. The key question is therefore not whether the innate surveillance system has a role in neurodegenerative disease, but what kind of role? Distinguishing whether innate surveillance has a pernicious or protective role in neurodegeneration has been problematic given its complex array of feed-forward and feed-back signaling mechanisms. For example it has been proposed that the role of innate surveillance in AD is in clearing amyloid beta plaques from the brain (Guillot-Sestler et al., 2015). This hypothesis is based on the observations that a reduction in anti-inflammatory cytokine (IL-10) signaling mitigates AD-like pathology, while over-expression reduces clearance in mouse models of cerebral amyloidosis (Guillot-Sestler et al., 2015). The innate surveillance system involves a mixture of anti- and pro-inflammatory cytokines, local and systemic actions, with neuroinflammation noted to have negative effects on neurogenesis and cognition (Ryan and Nolan, 2016). Genetic causes define the proximal defects and/or trigger molecules in pathways and on this basis identify the role of innate surveillance in neurodegeneration as causal.

The vast majority of AD cases are late age-at-onset and in these instances the most striking genetic contribution is a susceptibility locus on chromosome 19. A polymorphism within the *ApoE* gene is an indicator of age-at-onset (Saunders et al., 1993) and until recently it was assumed that variation in ApoE protein is the biochemical basis of this variation possibly through its impact on amyloid beta deposition (Masters et al., 2015). ApoE also affects the activity of innate surveillance signaling (Guillot-Sestler et al., 2015) providing an additional plausible mechanism for its variation impacting on AD pathogenesis. The *ApoE* gene is however located within a block of Linkage Disequilibrium raising the possibility that another gene also located within this block mediates AD susceptibility. One such gene is TOMM40 that also contains a polymorphism predictive of AD risk (Roses et al., 2010). TOMM40 is a component of the mitochondrial protein import complex and in this capacity is a key regulatory protein of mitochondrial function and the mitochondrial unfolded protein response mediated by ATFS-1 transport (see above and Gottschalk et al., 2015). Mitophagy plays a key role in Parkinson's disease pathogenesis, therefore variation in TOMM40 is also a plausible means by which polymorphism in the *ApoE/TOMM40* linkage disequilibrium block can have a causal role in AD.

#### Environmental

The vast majority of cases of neurodegeneration are thought to have environmental agents as their principle cause. Pathogen infection is likely to be one environmental cause. Sensitization is a key component of innate surveillance mediated by extracellular and systemic signaling molecules (*DAMPs* and *dAMPs*). Pathogens either directly or indirectly generate such signaling molecules, that the systemic component of innate surveillance then utilizes to communicate the presence of infection and primes the body to prepare for its potential spread. While such sensitization provokes host cells to then actively restrict infection, it also appears to increase susceptibility to other processes mediated by innate surveillance—including neurodegeneration. A very clear illustration of this phenomenon occurs following sepsis. Long-term cognitive impairment and functional disability are frequent consequences among survivors of severe sepsis (Iwashyna et al., 2010). The mechanism responsible is yet to be determined however the elevation of inflammation and resultant systemic sensitization has been proposed (Sharshar et al., 2014; Widmann and Heneka, 2014). Microglial activation plays a role in experimentally induced sepsis in a rodent model (Michels et al., 2015) and this is likely mediated by the *dAMP* HMG-B1 (Chavan et al., 2012; Valdes-Ferrer et al., 2013). Physical trauma, through head injury or ischemic stroke, are other factors thought to contribute to increased risk of neurodegeneration. Again HMG-B1 is a key mediator of immune mechanisms in ischemic stroke (Kim et al., 2006) and brain injury (Laird et al., 2014).

##### Inverse correlation between cancer incidence and neurodegenerative disease

While the ability to defend against pathogens is clearly essential for survival, the use of cell death as a defense strategy seems a very high biological cost to pay. It is therefore noteworthy that a clear inverse correlation has been found between the incidence of cancer and Alzheimer's disease (Driver et al., 2012; Musicco et al., 2013; Li et al., 2014). This inverse correlation with cancer incidence has also been observed for additional neurodegenerative disorders (notably PD and HD) in a large meta-analysis study (Catalá-López et al., 2014). These observations are consistent with the hypothesis that innate surveillance is the common mediator of both pre-cancerous cell elimination and neurodegeneration, explaining the trade-off between susceptibility to these conditions. A more sensitive innate surveillance system is more effective at detecting and eliminating dysfunctional, rogue cells that have accumulated somatic mutations otherwise leading them on a pathway of tumourigenesis. However this same sensitive innate surveillance system is more readily able to detect genetic and/or environmental danger and damage signals in the nervous system, leading to increased susceptibility to neurodegenerative disease. Conversely, a less sensitive innate surveillance system will confer a lower risk of neurodegeneration, but a higher risk that pre-cancerous cells will go undetected, and therefore lead to cancer.

#### Why are nerve cells sensitive to innate surveillance-mediated cell death?

The central nervous system has characteristics that may go some way to explaining its greater sensitivity to the consequences of innate surveillance activation. Peripheral tissues utilize the systemic component of innate surveillance to recruit specialized cells (primarily histiocytes) to the site of activation (Vanha-aho et al., 2015). These cells act in containment and resolution, however until elimination is achieved the combined cell mass can appear as a nodule referred to as a granuloma or inflammatory pseudotumors. While such structures appear common to many peripheral diseases they are very rare in the central nervous system (Tekkok et al., 2000; Hausler et al., 2003; Lui et al., 2009). This may be due to difficulty in attracting histiocytes into the CNS and/or that microglial cells fulfill such a role in the CNS. In any event the mechanics of resolving the cause of innate surveillance activation in the CNS appears to be distinct from that in peripheral tissues. Given the major role that microglial cells play in nerve cell maintenance it is noteworthy that microglial cell dynamics alter with age (Hefendhel et al., 2014), with a general reduction in functional capacity. Such changes may well confer a decreased ability to contain and resolve instances of innate surveillance activation and therefore mediate increased susceptibility to neurodegeneration with age.

##### If there is a common cause why are there are different diseases?

Differing susceptibilities are nothing new in genetic causes of disease. The same mutation in a single family can cause ALS or FTD or both (see—OMIM 105550). The same ADAR1 mutation can cause differing symptoms (Livingston et al., 2014). There are a large number and variety of innate surveillance components. It is likely that these components differ in their quantity and quality, even among differing nerve cell types, indeed such variation has already been reported (Applequist et al., 2002) with some of this variation likely due to the requirement for pathway components in normal developmental processes (Asakura et al., 2015). Therefore, disease-causing components are likely rate-limiting in some nerves, less so in others. Indeed neuronal plasticity may overcome single cell loss through alternative connections, but not the loss of the whole circuit. Finally, the strategy of adjacent cell sensitization that leads to foci of cell death, while working well in most tissues, may be ineffective in the aging brain with diminished microglial function.

### Future perspectives

The hypothesis stated here, that innate surveillance mediated cell death is the common cause of neurodegeneration, is a paradigm shift. This model heralds the exciting prospect of finally having a unifying view that accommodates all known data and reconciles some of the long-standing incompatibilities between previous models for neurodegenerative diseases. Until now the accumulating body of data in support of this innate surveillance hypothesis has been assembled from experiments that have not been specifically designed to test it. So it will now be important to directly challenge this hypothesis and its predictions. Numerous animal models and a vast quantity of clinical material are available for such targeted experiments and analyses. These experiments will need to build in the distinct possibility that innate surveillance is both protective early and pernicious late in neurodegenerative disease. Such information will be crucial for the design of clinical trials.

#### Detection and intervention—biomarkers for neurodegeneration

Reactive, presymptomatic biomarkers are needed, both to predict the onset of impending symptoms and to monitor the effectiveness of potential therapeutics in clinical trials. Given that innate surveillance activation is the proximal cause of neurodegeneration, it will be important to distinguish such biomarkers that are specific for neurodegeneration from those that possibly signal the activity of protective mechanisms (Zetterberg et al., 2013). Informative, quantifiable biomarkers for neurodegenerative disease are vital in assessing the effectiveness of interventions, particularly given the very long time frames involved for both age-at-onset and clinical progression for some of these diseases.

New targets for preventative therapies and/or drug interventions will be contingent not only upon defining key molecular processes and the identification of their rate-limiting steps, but also the window of time in which intervention is able to be effective. An intervention at the wrong time could impede a protective process rather than alleviate a pernicious one.

#### Genetic models of neurodegenerative disease

Distinguishing cause from consequence in the pathogenesis of disease can be problematic. Experimental animal models (both invertebrate and mouse) of the defined genetic causes of neurodegeneration in humans, are therefore a key strategy in establishing cause and effect relationships in the pathophysiology of neurodegenerative disease.

## Author contributions

RR conceived the hypothesis, initiated the assembly of supporting data, and wrote the first and final drafts following input from each of the co-authors. SR contributed material on the molecular and cellular components of inflammatory pathways, their roles in combating infection and their dysfunction in disease; intellectual contributions to both the formulation of the hypothesis and the form and content of the manuscript. LO contributed material on animals models, particularly *Drosophila;* commented and provided intellectual input into several manuscript drafts. DF conducted literature searches on RNA based pathology, particularly the distinction between “self” and “non-self” RNAs and contributed Figure 1; commented and provided intellectual input into several manuscript drafts. AS conducted literature searches on Parkinson's Disease and inflammatory pathways, assembled Figure 2, and detailed discussion of the contribution of mitochondrial dysfunction and mitophagy to neurodegenerative disease; commented and provided intellectual input into several manuscript drafts. ML conducted literature searches on Alzheimer's Disease and inflammation, and detailed discussion of the contribution of the membrane associated with the mitochondria (MAM) to Alzheimer's Disease. BB provided clinical insight into the occurrence of inflammation in the progression of neurodegenerative disease and the current state of presymptomatic markers; commented and provided intellectual input into several manuscript drafts.

## Funding

This research is funded in part by Project Grant (APP1069348) from the National Health and Medical Research Council of Australia and a grant from the National Ataxia Foundation (USA).

## Disclosures

RR has previously been a consultant for Glaxo-Wellcome (later GlaxoSmithKline), but is not currently and was not during the preparation of this manuscript.

### Conflict of interest statement

The authors declare that the research was conducted in the absence of any commercial or financial relationships that could be construed as a potential conflict of interest.

## Abbreviations

AGS: Aicardi-Goutieres Syndrome
ALS: Amyotrophic Lateral Sclerosis
DAMPs: Danger Associated Molecular Patterns
dAMPs: Damage Associated Molecular Patterns
FTD: Fronto-Temporal Dementia
PAMP: Pathogen Associated Molecular Pattern
PRR: Pattern Recognition Receptor
TLR: Toll-like Receptor
RLR: RIG-I-like Receptor.
