# Supplementary material for: The Enemy within: Innate Surveillance-Mediated Cell Death, the Common Mechanism of Neurodegenerative Disease
Source: Front Neurosci. 2016 May 10;10:193. doi: 10.3389/fnins.2016.00193 (PMC4862319; doi:10.3389/fnins.2016.00193)
Supplement: Supplementary file 1 [file Table1.pdf]

Supplementary Table and Abbreviations

Origin	DAMP / dAMP	PRR	References
MICROBIAL			
	Bacterial derived ligands	NLRC5, NLRP6, NLRP1	Kim *et al* 2016; Radian *et al* 2013; Thaiss *et al.* 2016; Yoneyama *et al* 2016
	e.g. peptidoglycan	NOD1	Kim *et al* 2016
	e.g. muramyl-dipeptide	NOD2, NLRP1	Kim *et al* 2016; Radian *et al* 2013
	e.g. rod protein	NLRC4, NAIP	Kim *et al* 2016; Radian *et al* 2013; Thaiss *et al.* 2016;
	e.g. flagellin	NLRC4, NAIP, TLR5	Goubau *et al* 2010; Radian *et al* 2013; Thaiss *et al* 2016
	DNA	AIM2, IFI16, TLR9, cGAS	Thaiss et al. 2016; Yoneyama *et al* 2016
	Viral RNA	RIG-1, MDA5, LGP2, TLR3, TLR7, TLR8, OAS	Thaiss et al. 2016; Yoneyama *et al* 2016
HOST CELLS			
	HMG-B1	TLR2, TLR4, TLR9, RAGE, TIM3	Ray *et al* 2015; Gelderblom *et al.* 2015; Kubes & Mehal 2012; Venereau *et al* 2015
	S100 proteins (calgranulins)	RAGE	Juranek *et al* 2015
	lipopeptide	NLRP7	Kim *et al* 2016
	mitochondrial-derived signals		
	e.g. mitDNA	NLRP3, TLR9	Wen *et al* 2014; Kubes and Mehal 2012
	e.g. cardiolipin	NLR3	Wen *et al* 2014
	e.g. mitochondrial transcription factor A	TLR9, RAGE	Venereau *et al* 2015
	e.g. Ca++	NLRP7, NLRP3	Kim *et al* 2016; Wen *et al* 2014
	e.g. cAMP depletion	NLRP3	Radian *et al* 2013; Wen *et al* 2014
	Guanylate-binding protein 5 (GBP5)	NLRP3	Radian *et al* 2013; Wen *et al* 2014
	Advanced glycation end products (AGEs)	RAGE	Ray *et al* 2015
	Heat shock proteins (HSPs)	TLR2, TLR4, CD14, CD91	Kubes and Mehal 2012; Gelderblom *et al* 2015
	ATPs	NLRP3, NLRP7, P2Y2, P2X7	Kim *et al* 2016; Radian *et al* 2015; Venereau *et al* 2015
	Double stranded RNA	TLR3, RIG1	Bernard *et al* 2012; Thaiss *et al* 2016
	Hyaluronan	TLRs	Gelderblom *et al* 2015
	F-actin	CLEC9A	Ahrens *et al* 2012; Venereau *et al* 2015
ENVIRONMENTAL			
	alum	NLRP3	Kim *et al* 2016
	asbestos	NLRP3	Thundyil and Lim 2015
	silica	NLRP3	Kim *et al* 2016
	anthrax	NLRP1	Kim *et al* 2016

Abbreviations

DAMP	= danger-associated molecular pattern
PRR	= pattern recognition receptor
NLRC	= NOD like receptor, CARD domain containing
NLRP	= NOD like receptor, pyrin domain containing
NOD	= nucleotide-oligomerization domain
NAIP	= NLR family, apoptosis inhibitory proteins
TLR	= toll-like receptor
AIM2	= absent in melanoma 2
IFI16	= interferon gamma inducible protein 16
cGAS	= cyclic guanosine monophosphate-adenosine monophosphate synthase
RIG-I	= retinioc acid inducible gene-I
MDA5	= melanoma differentiation factor 5
LGP2	= laboratory of genetics and physiology 2
OAS	= 2',5'-oligoadenylate synthetase
RAGE	= receptor of advanced glycation end-products
TIM3	= T-cell immunoglobulin domain and mucin domain 3
CD14	= cluster of differentiation antigen 14
CD91	= cluster of differentiation antigen 91
